# Lipid metabolism impairment in patients with sepsis secondary to hospital acquired pneumonia, a proteomic analysis

**DOI:** 10.1186/s12014-019-9252-2

**Published:** 2019-07-16

**Authors:** Narendra Kumar Sharma, Bianca Lima Ferreira, Alexandre Keiji Tashima, Milena Karina Colo Brunialti, Ricardo Jose Soares Torquato, Antonio Bafi, Murillo Assuncao, Luciano Cesar Pontes Azevedo, Reinaldo Salomao

**Affiliations:** 1 Division of Infectious Diseases, Escola Paulista de Medicina, Universidade Federal de São Paulo, Rua Pedro de Toledo, 669, 10th Floor, Sao Paulo, SP 04039-032 Brazil; 2 Department of Biochemistry, Escola Paulista de Medicina, Universidade Federal de São Paulo, São Paulo, SP 04023-900 Brazil; 3 Intensive Care Unit, Hospital São Paulo, Escola Paulista de Medicina, Universidade Federal de Sao Paulo, Sao Paulo, 04024-002 Brazil; 4 Intensive Care Unit, Hospital Israelita Albert Einstein, Sao Paulo, 05652-900 Brazil; 5 Intensive Care Unit, Hospital Sirio Libanes, Sao Paulo, 01409-001 Brazil; 6 Present Address: Department of Bioscience and Biotechnology, Banasthali Vidyapith, Banasthali Tonk, 304022 Rajasthan India

**Keywords:** Sepsis, Proteome, Hospital-acquired pneumonia, Lipid metabolism, Cholesterol

## Abstract

**Background:**

Sepsis is a dysregulated host response to infection and a major cause of death worldwide. Respiratory tract infections account for most sepsis cases and depending on the place of acquisition, i.e., community or hospital acquired infection, differ in etiology, antimicrobial resistance and outcomes. Accordingly, the host response may be different in septic patients secondary to community-acquired pneumonia and hospital acquired pneumonia (HAP). Proteomic analysis is a useful approach to evaluate broad alterations in biological pathways that take place during sepsis. Here we evaluated plasma proteome changes in sepsis secondary to HAP.

**Methods:**

Plasma samples were obtained from patients (n = 27) at admission and after 7 days of follow-up, and were analyzed according to the patients’ outcomes. The patients’ proteome profiles were compared with healthy volunteers (n = 23). Pooled plasma samples were labeled with isobaric tag for relative and absolute quantitationand analyzed by LC–MS/MS. We used bioinformatics tools to find altered functions and pathways. Results were validated using biochemical estimations and ELISA tests.

**Results:**

We identified 159 altered proteins in septic patients; most of them were common when comparing patients’ outcomes, both at admission and after 7 days. The top altered biological processes were acute inflammatory response, response to wounding, blood coagulation and homeostasis. Lipid metabolism emerged as the main altered function in patients, with HDL as a central node in the network analysis, interacting with downregulated proteins, such as APOA4, APOB, APOC1, APOL1, SAA4 and PON1. Validation tests showed reduced plasma levels of total cholesterol, HDL-C, LDL-C, non-HDL cholesterol, apolipoproteins ApoA1 and ApoB100, and Paraoxonase 1 in HAP patients.

**Conclusion:**

Proteomic analysis pointed to impairment of lipid metabolism as a major change in septic patients secondary to HAP, which was further validated by the reduced levels of cholesterol moieties and apolipoproteins in plasma. Our results stress the involvement of lipids in the pathogenesis of sepsis, which is in accordance with previous reports supporting the role of lipid moieties in pathogen toxin clearance and in modulating inflammatory responses.

**Electronic supplementary material:**

The online version of this article (10.1186/s12014-019-9252-2) contains supplementary material, which is available to authorized users.

## Introduction

Sepsis is defined as a life-threatening organ dysfunction caused by a dysregulated host response to infection [1]. It is a major cause of morbidity and mortality worldwide, with over 30 million estimated cases annually leading to 5.3 million potential deaths [2]. The burden of sepsis may be greater in developing countries [3], as illustrated by the findings that one-third of intensive care beds in Brazil were occupied by septic patients, with a mortality rate of 55.7% [4]. Sepsis may be secondary to community- or hospital-acquired infections, which differ in etiology, antimicrobial resistance and outcomes [5–7]. The respiratory tract is the most common site of infection, accounting for more than half of the cases of sepsis in intensive care units (ICU) [4, 8].

The pathogenesis of sepsis is complex and involves virulence factors from infectious microorganisms and the host defense immune system [9, 10]. Inflammatory and anti-inflammatory responses are triggered in sepsis, exposing patients to the potential harmful effects of inflammation or immunosuppression [11, 12]. Transcriptomics studies were pivotal in uncovering the broad derangements of the host following LPS exposure, trauma and sepsis [13–15]. Proteins are the actual players in biological systems; hence, proteome changes have been investigated in clinical and experimental sepsis revealing that biological pathways, such as inflammatory, acute phase response, coagulation, complement, mitochondrial energy metabolism, and oxidative stress pathways are altered at the protein level [16, 17].

We have recently reported that the proteomes of patients with sepsis secondary to community acquired pneumonia (CAP) are altered, in which cytoskeleton, cellular assembly, movement, lipid metabolism and immune responses are dysregulated [18]. Community and hospital acquired pneumonia are anticipated to present with different host responses during sepsis. A previous report evaluating a large cohort of CAP and HAP patients admitted to the ICU showed that patients with HAP presented with overexpressed genes involved in cell–cell junction remodeling, adhesion, and diapedesis, and an underexpressed type-I interferon signaling gene signature [19].

In this study, we evaluated proteome changes in septic patients secondary to HAP, in which we evaluated samples at admission and after 7 days of treatment and accordingly to outcomes, in survivors and nonsurvivors. Proteins were quantified using the iTRAQ method and bioinformatic approaches were used for identifying molecular functions, biological processes and pathways. Processes related to lipid metabolism were then identified as the most altered in the plasma of HAP patients.

## Material and methods

### Study design

In the present study, patients with sepsis secondary to HAP were selected and analyzed based on outcomes, such as septic survival and septic nonsurvival at hospital discharge. Hospital acquired pneumonia (HAP) occurs 48 h or more after admission and does not appear to be incubating at the time of admission; ventilator-associated pneumonia (VAP) is a type of hospital-acquired pneumonia that occurs more than 02 days of mechanical ventilation [20]. The patients’ plasma proteome profiles were compared with age and sex matched healthy volunteers.

### Sample collection

**Fig. 1:**
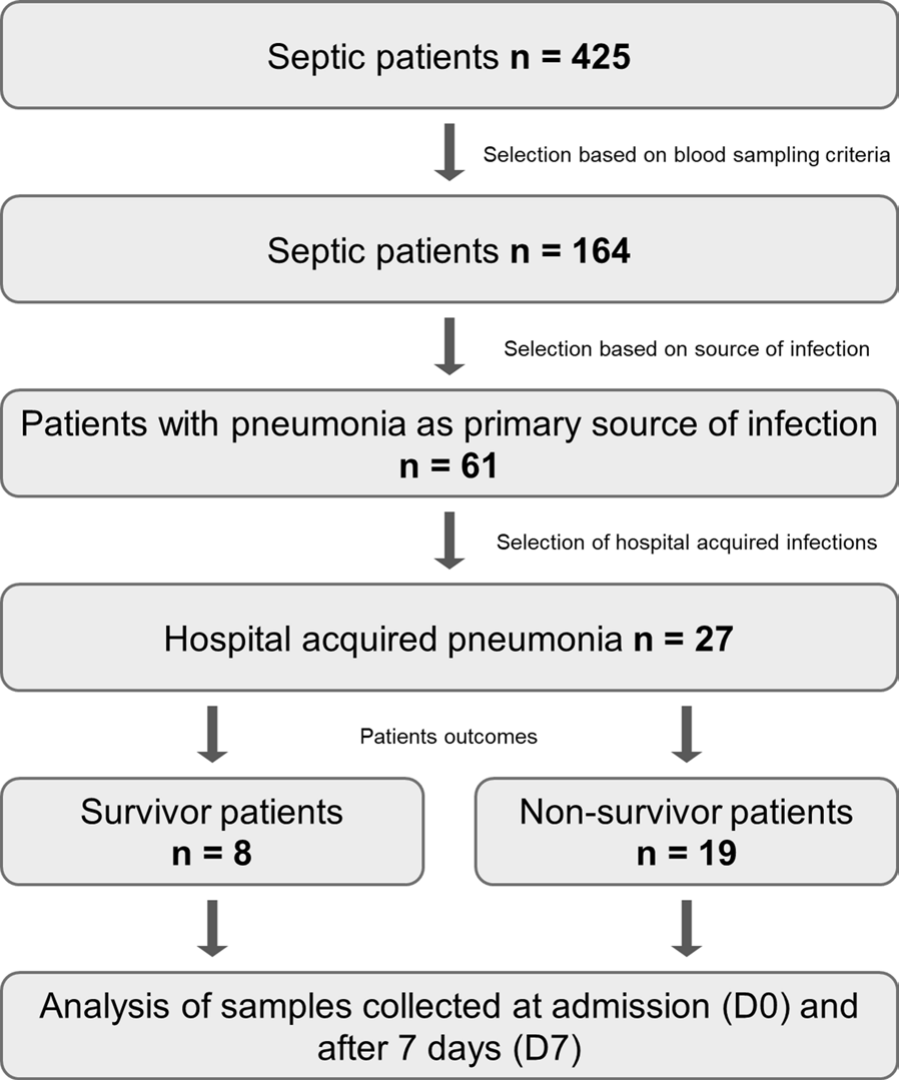
Schematic flow chart of the patient enrollment and selection. Patients admitted to intensive care units with severe sepsis and/or septic shock were selected based on criteria that included blood sampling, source and site of infection, and were assigned to groups according to their outcomes (survivors and nonsurvivors)

Blood samples were collected from healthy volunteers and from patients with severe sepsis/septic shock who were admitted into the ICUs of the participating hospitals after written informed consent was obtained from the participants or from their relatives. The prospective study was approved by the ethics committees of São Paulo Hospital (Study number 1477/06), Albert Einstein Hospital (Study number 07/549) and Sírio Libanês Hospital (Study number 2006/27). Patients with AIDS, immunosuppressive therapy or end stage chronic illness were excluded from the study. Fifty milliliters of blood was collected within 48 h of the first occurrence of organ dysfunction or septic shock (D0) and after 7 days of follow-up (D7). Plasma and blood cells were separated using a ficoll gradient (Ficoll-Paque PLUS; GE Healthcare Bio-Sciences AB, Uppsala, Sweden). A total of 425 septic patients were enrolled in the cohort, from which 27 septic patients, who had HAP as their primary source of infection and were older than 40 years of age, were selected for this study, 8 of whom survived and 19 of whom died during hospitalization (Fig. 1). Additionally, 23 healthy volunteers, who were matched by age and gender with the HAP patients, were selected from the 82 initially enrolled subjects for the study.

### Plasma sample processing

Plasma samples from septic patients were labeled as D0S and D7S, and D0NS and D7NS considering the day of collection and the outcomes, survivors (S) and non-survivors (NS). We estimated the protein content and pooled equal concentration of plasma protein from each individual sample to corresponding group before depletion. Healthy controls’ plasma samples were pooled in the same way.

Plasma albumin and immunoglobulins are major components (> 90%) of human blood and mask low abundant proteins. To unmask low abundant proteins, we depleted high abundant proteins using a proteome minor kit (BioRad, USA) and the depleted plasma samples were passed through a 3-kd filter with iTRAQ compatible buffer (Ab Sciex, USA). A total 100 μg of protein from all representative groups were transferred into separate tubes, and the volume was equalized with iTRAQ dissolution buffer. The cysteine disulfide bonds were reduced and alkylated using 50 mM TCEP and 200 mM methyl methanethiosulfate (MMTS). For protein digestion, 10 μg of trypsin was added to each vial, mixed and incubated at 37 °C overnight. The next day, the vial volume was reduced by SpeedVac and adjusted up to 30 μl using 1 M TEAB. A total of 60 μl of isopropanol was added to each iTRAQ reagent vial, mixed and quickly spun. The prepared iTRAQ reagent was added into the digested protein sample vial and incubated for 2 h at room temperature.

### Sample fractionation using SCX chromatography

To reduce the complexity, labeled peptides were fractionated using the SCX method. In brief, all sample vials were pooled into single vials and volume adjusted to pH < 2.7 using SCX-A buffer and 1 M hydrochloric acid. Then, the sample was applied to a PolySULFOETHYL A column and washed with 100% SCX-A at a rate of one ml per minute for 30 min. The labeled peptide mix was separated as described previously [18]. A total 20 fractions were collected and desalted using zip tip.

### LC–MS/MS analysis

Each fraction was applied to a nanoacquity UPLC nanoflow liquid chromatography system coupled with a Synapt G2 mass spectrometer (Waters, Milford, MA, USA). The fractions were further desalted in a trap column (180 µm × 2 cm, 5 µm, Waters, USA) at a flow rate of 8 µl/min for 5 min and then resolved on a C18 column (75 µm × 15 cm, 1.7 µm, Waters, USA) with an applied voltage of 3 kV. The peptides were separated using a linear gradient of 7–30% solvent B (90% acetonitrile in 0.1% formic acid) for 90 min with a flow rate of 250 nL/min. The MS data were acquired for the separated peptides in a data dependent manner from m/z 300 to 1600 Da with the three most abundant ions in the survey scan. For the MS/MS data, collision-induced dissociation (CID) mode was used with 1.5 s per spectra acquisition.

After data acquisition, raw files were processed with mascot distiller (Matrix Science, USA), and all processed MS–MS peak list files were merged with mascot daemon. Then, the merged file was searched against the UniProt database (20,120 entries of reviewed proteins in humans). The parameters included trypsin as a protease (allowed one missed cleavage), iTRAQ label at N-terminus and lysine residues, cysteine modifications by MMTS were specified as fixed modifications, and oxidation of methionine was specified as a modification variable. The precursor and product ion mass error tolerance were fixed at 20 ppm and 0.1 Da, respectively. The peptide and protein data were extracted using a high peptide confidence (*P* ≤ 0.05) and a minimum of 2 peptides were used for protein identification. The false discovery rate (FDR) was calculated using decoy database searches. Peptides identified at 1% FDR were used for protein identification. The results from the mascot server were loaded into isobaricQ for iTRAQ quantitation [21].

### Bioinformatic analysis of proteomics data

The identified proteins were converted to gene names/gene symbols to further analyze gene ontology, altered functions and pathways.

- Gene ontology annotations.

Gene ontology (GO) annotation was carried out using Toppgene suite, as described earlier [22]. In brief, a differentially expressed gene list was uploaded in the ToppFun section of Toppgene suite with an FDR B&Y correction and a *P* value cut off 0.05. The resulting file, which contained altered molecular functions and biological processes, was analyzed.

b. Function and pathway analysis.

The gene list of identified proteins was uploaded into the Ingenuity pathway knowledge database (IPA) and the fold change cut off was set at ± 1.3 for further functional, pathway and regulatory network analyses. The significant altered functions and signaling pathways (*P* < 0.05) were included.

### Plasma protein quantitation and biochemical estimations

To validate the proteomics data, we selected altered plasma proteins and cholesterol fractions for biochemical assays in individual samples from patients and healthy volunteers. For analysis, the COBAS c311 automated system was used. Cholesterol fractions and triglycerides were estimated, per the manufacturer’s protocol, using enzymatic and colorimetric methods (CHOL HICo Gen.2, HDL-C Gen.3 and TRIGL, Roche, USA). Plasma lipoprotein, apolipoprotein A-1 and apolipoprotein B were quantified, per the manufacturer’s protocols, using immunoturbidimetric methods (Tina-quant Lipoprotein (a) Gen.2, Tina-quant Apoliprotein A-1 ver.2 and Tina-quant Apoliprotein B ver.2, Roche, USA).

PON-1 and haptoglobin plasma levels were quantified by ELISA. PON-1 was measured with a human total PON1 DuoSet^®^ IC (DYC5816-2, R&D Systems, USA) and haptoglobin with a human haptoglobin immunoassay Quantikine^®^ ELISA (DHAPG0, R&D Systems, USA), following the manufacturer’s instructions.

### Statistical analysis

The Shapiro–Wilk test was used to evaluate normality. For clinical data analysis, Fisher’s exact test was used for categorical variables and the unpaired *t* test was used for numerical variables. Differences in plasma levels of lipids and lipoproteins were analyzed by one-way ANOVA with the Bonferroni post hoc multiple comparison test. For PON-1 and haptoglobin ELISA analyses, the Kruskal–Wallis test was used to evaluate differences between patients and healthy volunteers. All differences were considered significant when a *P* value was ≤ 0.05. Analyses were performed using Graph Pad Prism 6 (GraphPad Software, Inc., USA).

## Results

### Clinical data

Demographic and clinical data from patients are described in Table 1. The average age of the septic patients was 62 years old and 70% of them were males. Most patients acquired pneumonia prior to ICU admission, presented with septic shock, and cardiovascular and respiratory dysfunctions were their main organ dysfunctions. Comparisons between the patients who survived and those who did not survive were not significantly different regarding the percentage of septic shock, severity scores, organ dysfunction or underlying conditions.

**Table 1:** Clinical variables and demographic data from septic patients

	Control (n = 23)	Sepsis (n = 27)	Survival (n = 8)	Non-survival (n = 19)	*P* value^a^ S × NS
*Demographic data*					
Age, mean ± SD, year	65 ± 14.6	62.4 ± 12.8	60 ± 15.8	63.4 ± 11.6	0.5357
Sex (% of male)	60.9	70.4	87.5	63.2	0.3645
Place of acquired infection (%)					
Prior to ICU	NA	77.8	87.5	73.68	0.6334
ICU acquired	NA	22.2	12.5	26.32	
*Severity of disease*					
Septic shock (%)	NA	77.8	87.5	73.68	0.6334
Severity scores, mean ± SD					
Apache II	NA	17.1 ± 6.5	15.6 ± 7.6	17.7 ± 6	0.4483
SOFA	NA	8.3 ± 2.9	8.0 ± 2.9	8.4 ± 2.9	0.7709
Delta-SOFA (D3-D0)	NA	(−) 0.4 ± 3.1	(−) 1.9 ± 3.4	0.7 ± 2.8	0.0666
Organ dysfunction (%)					
Cardiovascular	NA	88.9	87.5	89.5	1
Renal	NA	37	37.5	36.8	1
Respiratory	NA	85.2	75	89.5	0.5583
Hematological	NA	11.1	0	15.8	0.5323
Hepatological	NA	33.3	25	36.8	0.6758
Central nervous system	NA	44.4	50	42	1
*Underlying conditions*					
Chronic comorbidity (%)					
AIDS	NA	0	0	0	–
COPD	NA	15.4	12.5	16.7	1
Diabetes	NA	30.8	12.5	38.9	0.3602
Chronic renal disease	NA	7.7	0	11.1	1
Cardiovascular insufficiency	NA	11.5	0	16.7	0.5292
Drugs (%)					
Corticosteroids	NA	37	50	31.6	0.4147

*S* survivor, *NS* non-survivor, *NA* not applicable, *SOFA* Sequential [Sepsis-related] Organ Failure Assessment, *COPD* chronic obstructive pulmonary disease

^a^Fisher’s exact test or unpaired t-test were applied to determine the *P* value when comparing survival and non-survival groups

### Most altered proteins were common in the patients’ groups, despite their outcomes

**Fig. 2:**
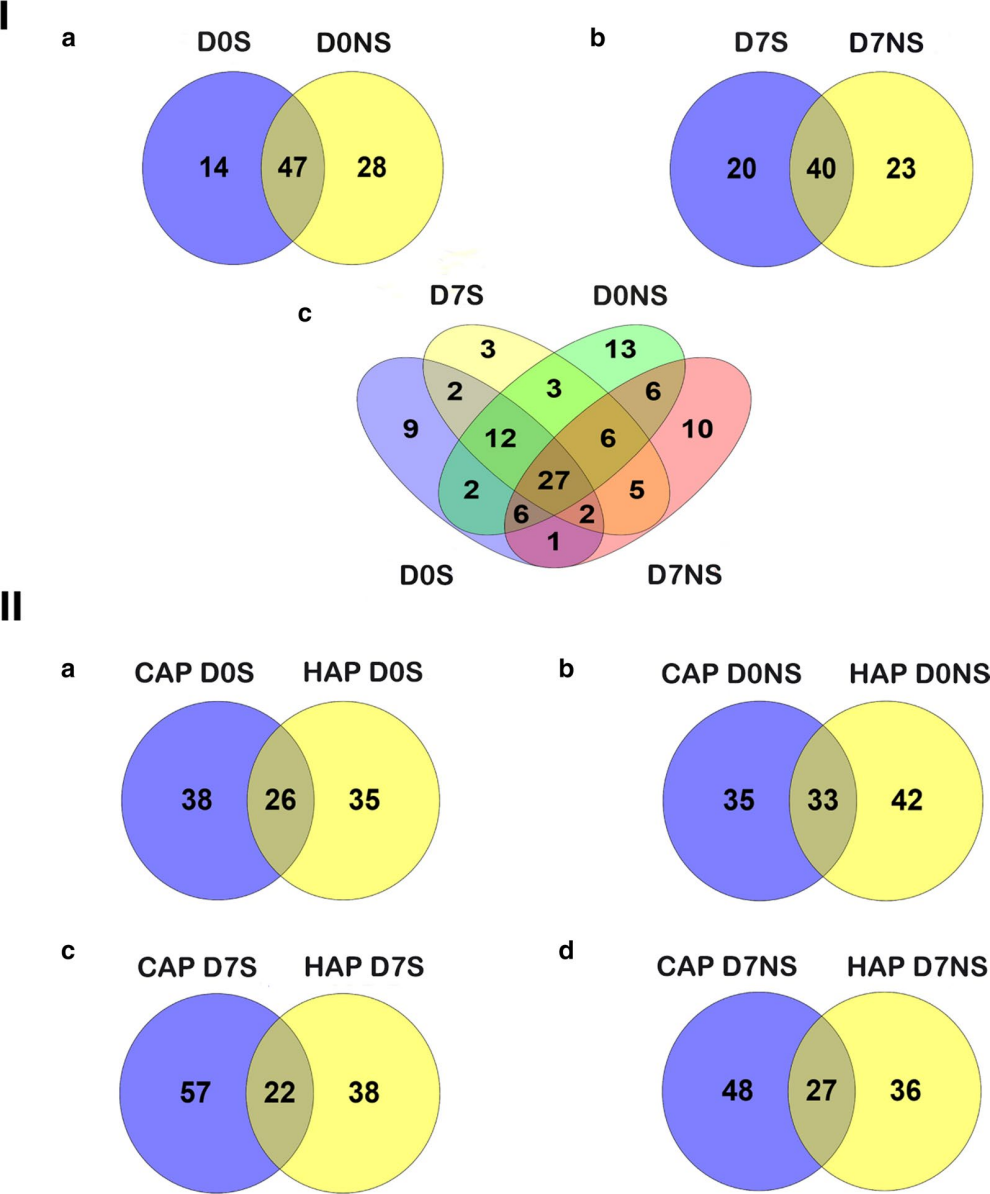
A Venn diagram showing differential proteome expression between the septic patient groups. **I** shows the differential protein expression levels in survivors and nonsurvivors at admission (**I**a) and at D7 (**I**b), and the differential expression levels at D0 and D7 in survivors and in nonsurvivors (**I**c). **II** demonstrates the differential expression levels between community-acquired pneumonia and hospital-acquired pneumonia (a–d). CAP, community-acquired pneumonia; HAP, hospital-acquired pneumonia. D0S and D7S, admission and follow-up samples in survivors, and D0NS and D7 NS, admission and follow-up samples in nonsurvivors

Using a quantitative proteomics approach, we selected 159 proteins for analysis after removal of albumin, immunoglobulin and their isoforms from a total of 220 proteins. At admission, 61 and 75 proteins were differentially expressed in the septic survivors and nonsurvivors, respectively, and 60 and 63 proteins were identified after 7 days in these groups when compared with healthy volunteers (Additional file 1). Venn diagram analysis enabled us to identify 14 proteins that were exclusively altered in survivors and 28 proteins in nonsurvivors, while 47 proteins were common in both groups at admission. Similarly, 20 proteins were identified exclusively in the survivors, 23 proteins were identified in the nonsurvivors and 40 proteins were identified that were common in both groups after 7 days (Fig. 2I, Additional file 2). We also analyzed the differentially expressed proteins from our previous community acquired pneumonia (CAP) results [18] and found that the majority of the proteins were different between HAP and CAP. We found that, at admission, 26 proteins were common, while 38 and 35 proteins were specific to CAP and HAP survivors, respectively. Similarly, 33 proteins were common, while 35 and 42 proteins were specific to septic nonsurvivors in the CAP and HAP groups, respectively. Furthermore, after 7 days, 22 and 27 proteins were common in the survivor and nonsurvivor groups, while 57 and 38 were specific to the CAP and HAP survivors, and 48 and 36 proteins were specific to the CAP and HAP nonsurvivors, respectively (Fig. 2II, Additional file 2).

### Identification of altered pathways by gene ontology and ingenuity pathway analysis

**Fig. 3:**
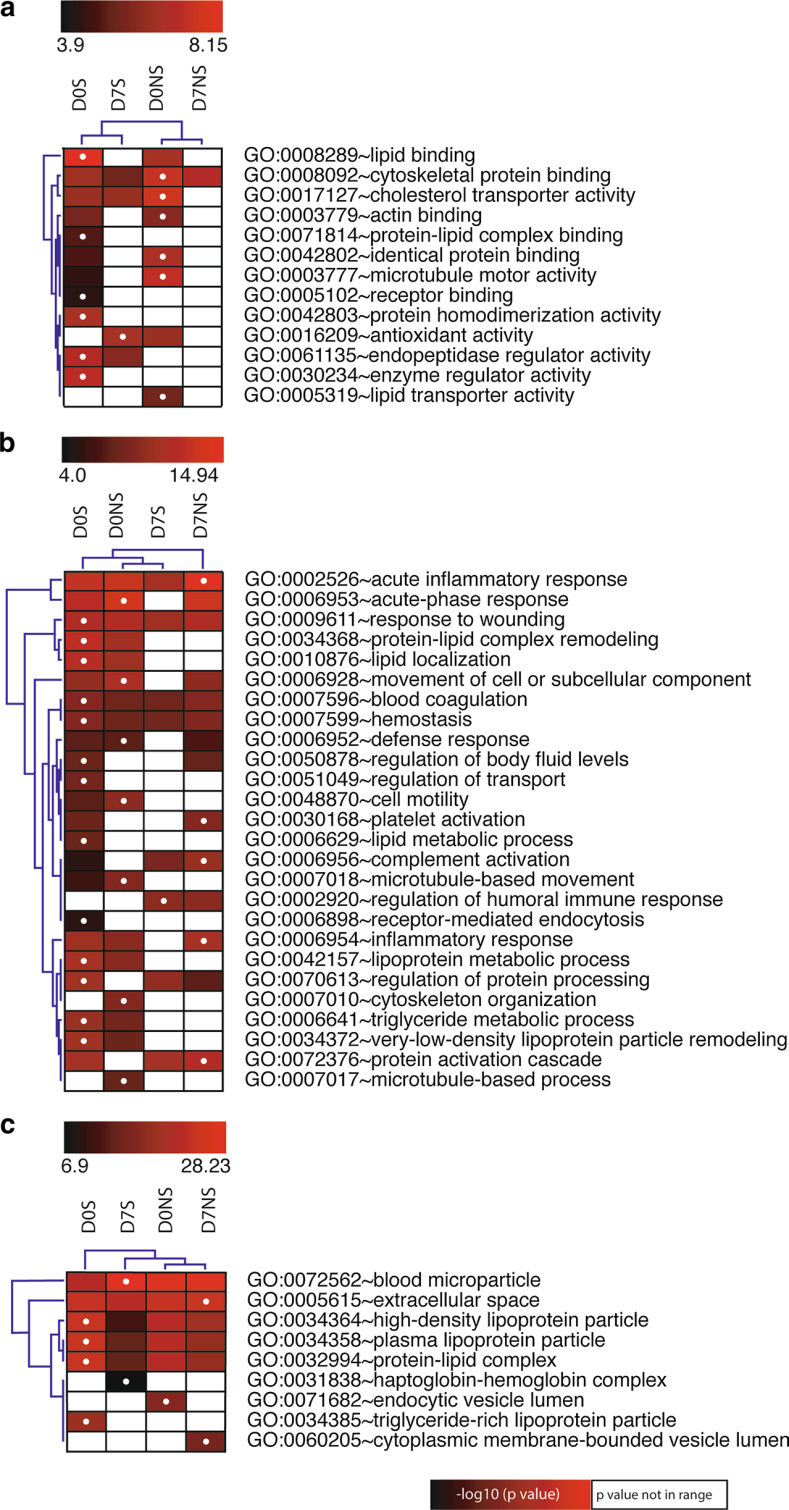
Gene ontology annotations for the identified differentially expressed proteins. Altered molecular functions (**a**), biological processes (**b**) and cellular components (**c**) in septic survivors and nonsurvivors at admission and after 7 days. The altered functions are represented as − log10 (*P* value) with the highlighted dots representing the group with maximum changes for a function. The white squares represent *P* values that were not included in the range selected for each analysis

The gene names that corresponded with the differentially expressed identified proteins were generated for GO analysis. The top altered molecular functions included lipid binding and cytoskeleton protein binding in the survivors and nonsurvivors at admission (Fig. 3a). The top altered biological processes were acute inflammatory response, response to wounding, blood coagulation and homeostasis in all septic patients, regardless of outcome or time of enrollment. We found that lipid localization, lipoprotein metabolic process, triglyceride metabolic process, VLDL particle remodeling and cell motility were altered in septic patients at admission, while humoral immune response was found after 7 days (Fig. 3b). When analyzing cellular components, the proteins were mostly localized in blood microparticles, extra cellular space, HDL particles, plasma lipoprotein particles and protein lipid complexes (Fig. 3c).

Similar results were obtained when analyzing canonical pathways with IPA. Pathways such as LXR/RXR activation, FXR/RXR activation (both related to lipid homeostasis [23]), acute-phase response signaling and coagulation were found to be altered in all patient groups (Additional file 3).

**Fig. 4:**
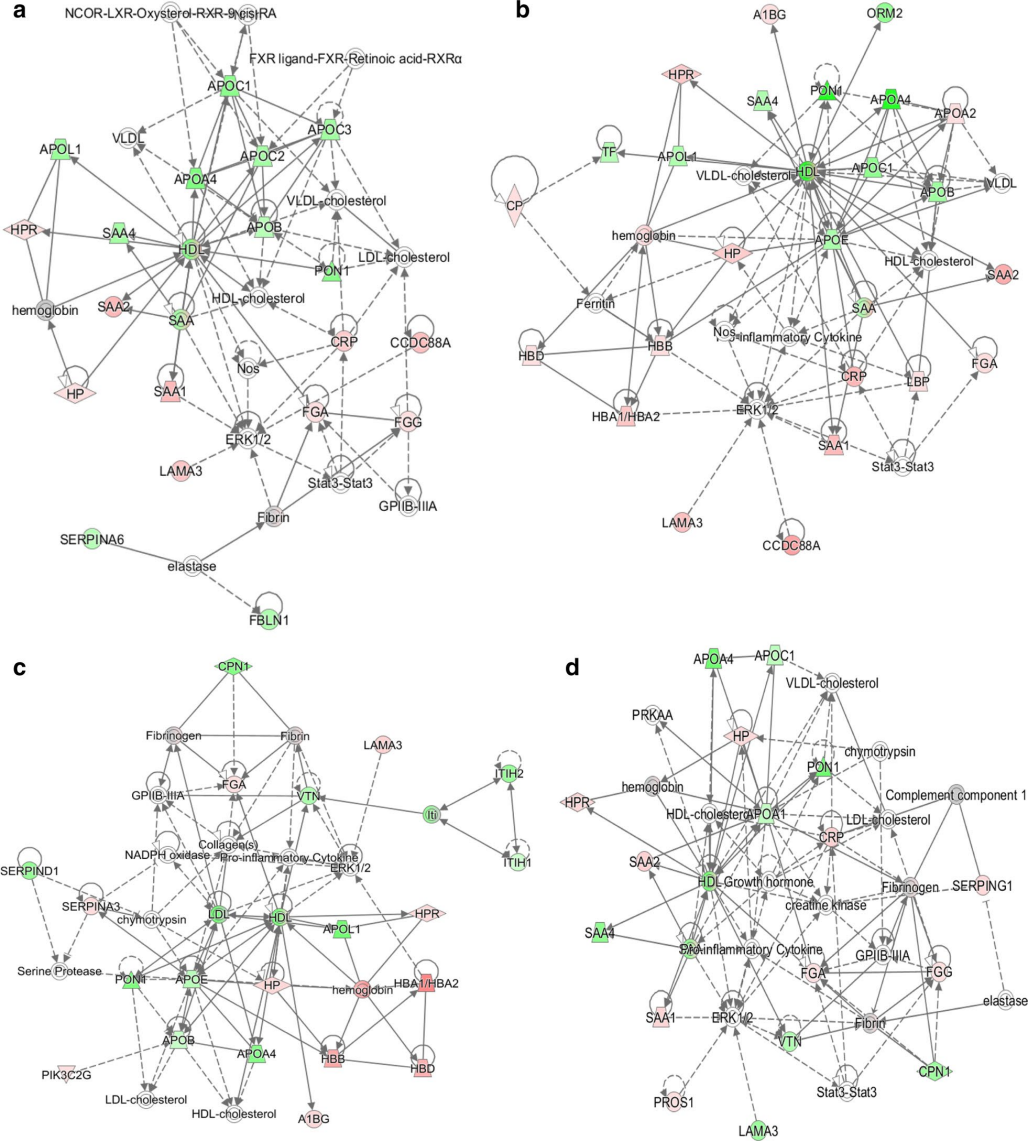
Protein-protein interactions and functional networks. The red color represents upregulation and the green color represents downregulation. **a**–**d** corresponds to the D0 survivors, D0 nonsurvivors, D7 survivors and D7 nonsurvivors, respectively

The top IPA scored disease and functions in the septic patients at admission were lipid metabolism, molecular transport and small molecule biochemistry (Additional file 4). Interaction network analysis of those functions show that HDL was a central node protein in the network, which interacts with downregulated proteins, such as APOA4, APOB, APOC1, APOL1, SAA4 and PON1. Upregulated proteins, such as CRP, HP, SAA1, FGA and LAMA3 also interacted with HDL, directly or indirectly (Fig. 4a, b). After 7 days, different functions were top scored, but HDL remained a central node in the interaction network, both in the survivor and nonsurvivor groups (Fig. 4c, d, Additional file 4). Finally, functions related to lipid metabolism were impaired in all patients (Additional file 5).

### Altered proteins related to lipid metabolism and other biological functions

**Fig. 5:**
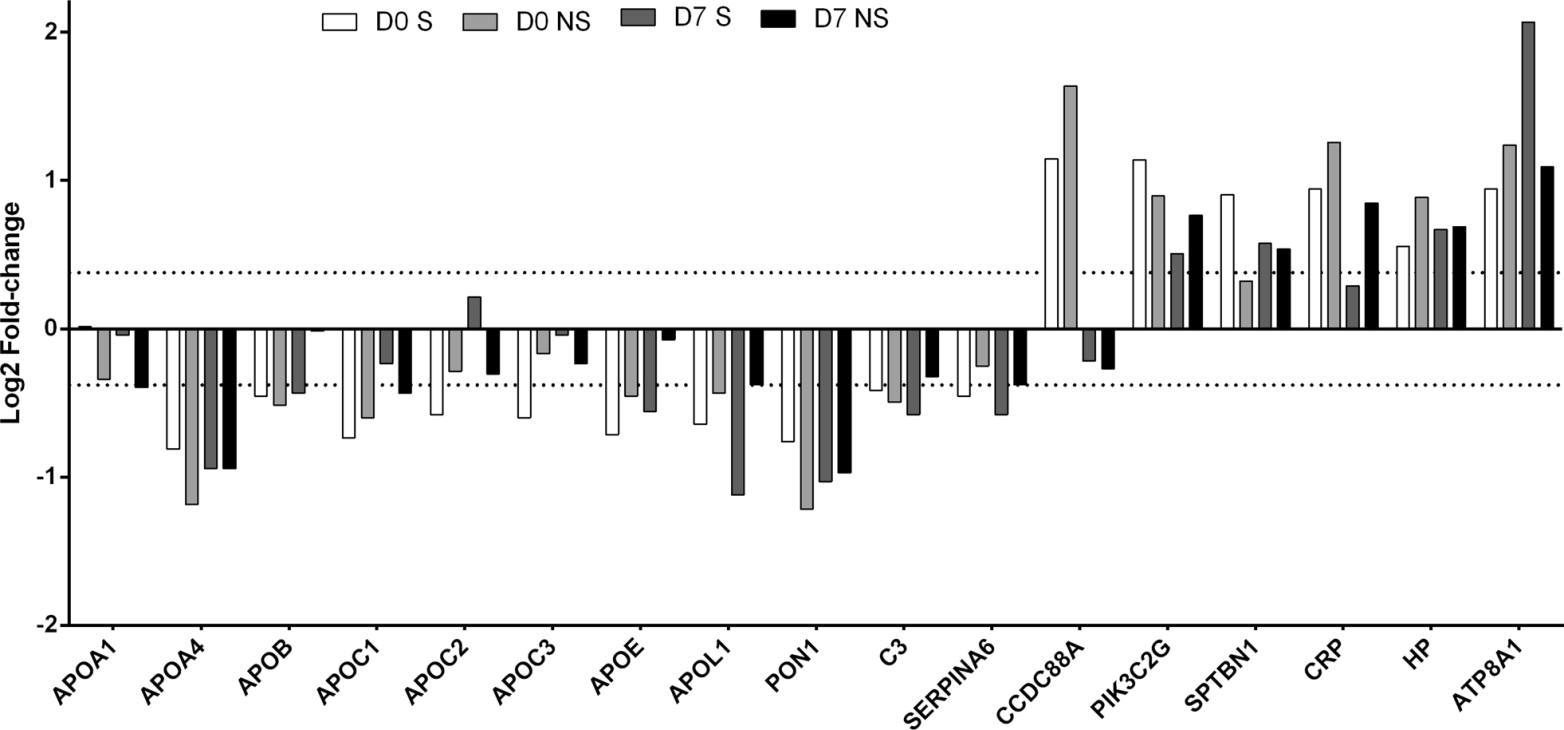
Expression of lipid metabolism related proteins. The bar chart represents log_2_-fold-changes (patients vs. healthy volunteers) of altered proteins related to lipid metabolism. The dashed line represents the fold-change cut-off (log_2_-fold-change │0.3785│, corresponding to fold-change ± 1.3)

Proteomics analysis enabled us to identify several apolipoproteins that act on lipid transportation in septic patients. We identified decreased levels of Apo AI, Apo AIV, Apo B100, Apo CI, Apo CII, Apo CIII, Apo E and Apo L in septic patients. In addition to apolipoproteins, we identified other altered lipid binding proteins. Serum paraoxonase 1 (PON1), complement (C3) and corticosteroid-binding globulin (SERPINA6) were lower in patients than in controls. The expression levels of phosphatidylinositol 4-phosphate 3-kinase C2 domain-containing subunit gamma (PIK3C2G), spectrin beta chain, nonerythrocytic 1 (SPTBN1) and C-reactive protein (CRP) were higher in septic patients than in controls at day 0 and day 7, while girdin (CCDC88A) was only higher at day 0. Haptoglobin (HP), which is related to both lipid metabolism and inflammation, and phospholipid-transporting ATPase IA (ATP8A1) were expressed at higher levels in the septic patients (Fig. 5). These proteins are involved in several functions, such as lipid homeostasis, lipoprotein metabolic processes, lipid transport, lipid localization, lipid catabolic processes, cholesterol transport, cholesterol homeostasis, cholesterol efflux, high-density lipoprotein particle remodeling and very-low-density lipoprotein particle remodeling.

In addition to lipid metabolism, proteins related to acute phase response were also altered in the patients. In addition to CRP and HP, alpha-1-antichymotrypsin (SERPINA3) and serum amyloid A-1 and A-2 proteins (SAA1 and SAA2) were upregulated in patients at admission and after 7 days of follow-up, while serum amyloid A-4 protein (SAA4) and prothrombin (F2) were downregulated. These and other dysregulated proteins are related to inflammation (SERPINA3, SAA1, SAA2, SAA4, HP, C3, C6, C8B, F2, CRP, APOC3, APOE and KNG1), complement (C3, C6, C8B and CRP) and coagulation (SAA1, KIF15, FGA, C3, APOB, FGG, APOE, F2, TTN, SERPIND1 and KNG1). The expression data are available in Additional file 1.

To confirm the obtained results, we estimated the plasma levels of total cholesterol, HDL-C, LDL-C, triglycerides, ApoA-I, Apo B and lipoproteins in individual samples from patients and healthy volunteers. We found that the total cholesterol, non-HDL cholesterol, HDL-C, LDL-C, Apo AI and Apo B levels were significantly decreased in the septic patients. No significant changes were found in the total triglyceride and lipoprotein levels. Also related to lipid metabolism, the levels of serum paraoxonase (PON1) were decreased in the septic patients. In contrast, the HP levels tended to be increased in the septic patients (Table 2).

**Table 2:** Estimation of lipid moieties and proteins in blood plasma

Plasma level median (IQR)	Control (n = 10)	Survivor patients (n = 6)		Non-survivor patients (n = 11)	
		D0	D7	D0	D7
Total cholesterol (mg/dL)	198	90.5*	110.5*	105*	104*
	(176.5–245.5)	(79–121.5)	(103–145.5)	(86–125)	(91–150)
HDL-C (mg/dL)	54.5	20*	33.5*	23*	20*
	(45.8–59.3)	(7.8–33.5)	(17.8–42.5)	(21–35)	(12–48)
LDL-C (mg/dL)	121.5	51*	68*	56*	59*
	(89–161.3)	(39.8–79)	(56.3–80)	(52–68)	(52–83)
Non-HDL-C (mg/dL)	148	70*	86*	79*	86*
	(121.8–190.3)	(54–99.5)	(70–109.3)	(72–97)	(77–103)
Triglycerides (mg/dL)	144	89.5	103	125	127
	(115–184.5)	(64.5–165.3)	(67.5–200)	(85–179)	(106–177)
APO A-I (mg/dL)	151.5	65*	92*	67*	67*
	(139.8–177.5)	(45.8–88.3)	(58.8–109.8)	(63–88)	(49–91)
APO B (mg/dL)	104	56.5*	72.5*	71*	77
	(85.5–141.5)	(50.5–73)	(68.3–78.5)	(48–86)	(71–112)
Lipoproteins (mg/dL)	15.5	5.8	5.9	6.5	13.2
	(8.6–26.9)	(3.27–25)	(4.12–31.6)	(4.9–14.9)	(5.8–36.8)
PON 1 (ng/mL)^#^	210.2	106.3*	111.9*	110.2*	113.8*
	(170.1–313.7)	(78.9–141.5)	(83.6–189.4)	(93.5–183.6)	(98.5–174.9)
Haptoglobin (mg/mL)^#^	0.98	1.6	1.4	1.3	1.5
	(0.7–1.3)	(0.9–2.1)	(0.8–1.7)	(0.9–1.7)	(1.2–1.6)

*P ≤ 0.05 when comparing patients to healthy volunteers by Kruskal–Wallis test or one-way ANOVA with Bonferroni’s post hoc multiple comparison test

^#^PON 1 measurements were performed in 11 individuals in control group, 11 in survivors and 12 in non-survivors group; haptoglobin measurements were performed in 8 controls, 4 survivors patients and 10 non-survivors patients

## Discussion

Sepsis is a major cause of death in ICUs and the respiratory tract is the main primary infection site in septic patients. We previously evaluated the plasma proteome of patients with sepsis secondary to community-acquired pneumonia [18]. Here, we reported on plasma proteome alterations in patients with sepsis secondary to hospital-acquired pneumonia.

Different bioinformatics analyses revealed changes in acute phase response, inflammatory response and blood coagulation in this septic patient cohort, as previously described [24–26]. Nevertheless, lipid metabolism processes emerged as the main changes in the septic patients compared with healthy volunteers. These observations do not differ very much from what we found in the CAP patients [18], despite the fact that most altered proteins in the HAP patients were distinct from the CAP patients. A recent study that compared host responses to CAP and HAP described similar genomic alterations in both clinical groups, despite differences in the pathogens and conditions that lead to infection in each case [19].

HDL was found to be a central node in the interaction network analysis in all patient groups, regardless of outcome or the time of enrollment. It is well known that, depending on the protein and lipid composition, HDL can develop an anti-inflammatory or an inflammatory profile [27, 28]; it is also well known that both infection and sepsis decrease the plasma levels of HDL in patients [29, 30]. HDL with an inflammatory profile is related to SOFA score [31] and elder patients with sepsis were reported to present with a lower cholesterol efflux capacity, which is the main function of HDL, and a higher HDL inflammatory index [32].

In our proteomics results, we found decreased expression of PON1 and the apolipoproteins related to HDL (APO A1, APO C and APO E), and increased levels of HP and SAA1/SAA2. Additionally, we observed by biochemical analysis that the total cholesterol, HDL, APO A1 and PON 1 levels were decreased in the patients. These findings are similar to our previously reported changes in patients with sepsis and CAP [18] and are in accordance with the literature, which points to HDL turning into a pro-inflammatory mediator in septic patients. The reduction in APO A1 levels and increase of SAA levels is a characteristic change in the so-called acute-phase HDL [28] and was previously observed in sepsis [33]; furthermore, decreased APO A1 levels are related with mortality in septic patients [34].

Paraoxonase 1 is a component of HDL that acts as an antioxidant enzyme [35]. The reduction of PON1 in HDL is related to inflammatory conditions [35] and it was reported that PON1 has lower activity in septic patients, which is normalized after recovery [36]. Additionally, nonsurvivor patients presented with even lower activities than those who survived [37]. In addition, we found increased expression of HP in HAP patients, although this result was not significant in the validation step. However, data in the literature corroborate our observation. For example, HP was found to be a good biomarker for sepsis development in trauma patients [38]. HP is a scavenger of free hemoglobin [39] and it may play a protective role in septic patients, as they can present with elevated levels of cell-free hemoglobin [40]. In contrast, HP when associated with HDL can contribute to pro inflammatory responses [41]. It was described that HP binding to ApoA1 impairs HDL function and that peptides that displace HP from ApoA1 can reverse this phenotype [42]. Additionally, HP gene polymorphisms were related to altered levels of LDL and CRP, and the ApoA1/ApoA2 ratio in plasma [43]. Nevertheless, binding of HP to ApoA1 during an acute phase response can protect ApoA1 from oxidative damage [44].

Sepsis and systemic inflammation decrease not only the levels of HDL but also promote hypocholesterolemia, with lower levels of total cholesterol and LDL [28]. We observed reduced levels of total cholesterol in HAP patients, which was similar to what was observed in CAP patients. It was reported that hypocholesterolemia is related with severity and that cholesterol levels increase during convalescence in severely injured patients [45]. We also observed decreased levels of Apo B, LDL and non-HDL cholesterol in HAP patients, while in our previous work with CAP, the levels of these plasma components were not significantly lower than controls [18].

Similar to HDL, LDL has an important role in neutralizing pathogen toxins, such as LPS [28]. Low LDL levels were associated with the presence of fever and sepsis in hospitalized patients [46] and with long-terms rates of sepsis [47]. Additionally, it was observed that even with LDL levels below normal, septic patients present with higher levels of oxidized LDL, which is pro inflammatory [48]. The major apolipoprotein of LDL is Apo B, which was reduced in septic patients in our proteomic results. Apo B levels was reported to be increased after *Escherichia coli* sepsis in an experimental model [49]; in human sepsis, LPS-binding protein—that interacts with ApoB was found to be associated with LDL and VLDL particles [50]. In this context, our results point to LDL as contributing to inflammation and with impaired scavenger capacity. It is noteworthy, however, that while reduced LDL production is related to a complicated prognosis, LDL clearance can improve survival [51].

In conclusion, our proteomic study stresses the lipid metabolism as a major altered function in the plasma of patients with sepsis secondary to hospital acquired pneumonia, which is in accordance with previous reports supporting the role of lipid moieties in pathogen toxin clearance and in modulating inflammatory responses. Interestingly, HDL-C and cholesterol levels have been associated with risk of nosocomial infection acquisition [52]. These results reinforce the importance of lipid metabolism in sepsis pathogenesis and as a possible therapeutic target.

Our study has some limitations. We used pools of samples to run proteomics for the different groups of patients, D0S and D7S, and D0NS and D7NS, and healthy volunteers. The characteristics and the limited number of tags available for quantification in the iTRAQ protocol favours the conduction of the experiments with pooled samples. Several other clinical proteomics studies with iTRAQ were performed with pooled samples [53]. However, we are aware of the limitations of using pooled samples. To overcome these limitations, for validation, we used individual samples for representative groups. By choosing healthy volunteers as controls, it is not possible to differentiate the changes in plasma proteome that are specific for the septic patients secondary to HAP from those that take place in another critical illnesses. Furthermore, some underlying conditions not covered in our survey could be present in patients and influenced the proteome changes we are reporting.

## Additional files

**Additional file 1.** Proteomics Quantification - Raw and processed data.

**Additional file 2.** The details of the common and differentially expressed proteins in different groups corresponding to Venn diagram. CAP, community-acquired pneumonia; HAP, hospital-acquired pneumonia. D0S and D7S, admission and follow-up samples in survivors. D0NS and D7 NS, admission and follow-up samples in non-survivors.

**Additional file 3.** IPA canonical pathway analysis in septic patients. A refers to altered canonical pathway in D0 survivors; B refers to D0 non-survivors; C refers to D7 survivors; and D refers to D7 non-survivors. Enriched canonical pathways were identified from the IPA library using Fisher’s exact test adjusted for multiple hypothesis testing with the Benjamini- Hochberg correction.

**Additional file 4.** Functional protein interaction network for septic patients. The bold symbols represent the proteins identified in our study with their functions. The green arrow shows decreased expression and red arrow show increased expression compared with healthy volunteers.

**Additional file 5.** Functional analysis curated by Ingenuity Pathway Analyses. Prediction of altered functions based on activation Z-score. A score lower than -2 or higher than 2 predicts decreased or increased activation for each function. S: survivor. NS: non-survivor.

## Data Availability

All data generated or analyzed during this study are included in the manuscript and the additional files. Any further information is available from the corresponding author on request.

## Abbreviations

CAP: community-acquired pneumonia
HAP: hospital-acquired pneumonia
iTRAQ: isobaric tag for relative and absolute quantitation
TCEP: tris(2-carboxyethyl)phosphine
TEAB: triethylammonium bicarbonate
IPA: ingenuity pathway analysis
SOFA: sequential [sepsis-related] organ failure assessment
