# Supplementary material for: Lipid metabolism impairment in patients with sepsis secondary to hospital acquired pneumonia, a proteomic analysis
Source: Clin Proteomics. 2019 Jul 16;16:29. doi: 10.1186/s12014-019-9252-2 (PMC6631513; doi:10.1186/s12014-019-9252-2)
Supplement: Supplementary file 2 — Additional file 2. The details of the common and differentially expressed proteins in different groups corresponding to Venn diagram. CAP, community-acquired pneumonia; HAP, hospital-acquired pneumonia. D0S and D7S, admission and follow-up samples in survivors. D0NS and D7 NS, admission and follow-up samples in non-survivors. [file 12014_2019_9252_MOESM2_ESM.docx]

**Additional File 2. The details of the common and differentially expressed proteins in different groups corresponding to Venn diagram.** CAP, community-acquired pneumonia; HAP, hospital-acquired pneumonia. D0S and D7S, admission and follow-up samples in survivors. D0NS and D7 NS, admission and follow-up samples in non-survivors**.**

| **I A. Comparison between survivors and non-survivor’s day 0 in HAP (D0 S x D0 NS)** | | |
| --- | --- | --- |
| 14 proteins included exclusively in  "D0 S": | 28 proteins included exclusively in "D0 NS": | 47 common proteins in "D0 S" and "D0 NS": |
| FGG | CP | C3 |
| APOC2 | TF | SAA1 |
| APOC3 | HBB | SAA2 |
| FBLN1 | A1BG | FGA |
| PZP | GSN | APOB |
| C6 | HBD | SERPINA3 |
| KNG1 | CPN1 | APOE |
| C8B | APOA2 | LRG1 |
| SERPINA6 | HBA1 | F2 |
| PNPLA8 | DNAH17 | PON1 |
| TTN | CEP350 | HP |
| SPTBN1 | ELMO1 | APOA4 |
| TPR | LBP | CRP |
| MUC16 | FLNB | HPR |
|  | PRPF8 | KRT76 |
|  | ORM2 | ITIH2 |
|  | LYZ | SERPIND1 |
|  | KIF7 | APOL1 |
|  | PCNT | APOC1 |
|  | MKI67 | DST |
|  | OBSCN | PIK3C2G |
|  | LTF | HLTF |
|  | NIPBL | PLCH1 |
|  | SPAG17 | DNAH8 |
|  | SPTBN5 | SAA4 |
|  | WDR87 | LRRK2 |
|  | EVPL | KMT2C |
|  | MCF2L2 | DNAH5 |
|  |  | JAK1 |
|  |  | FRAS1 |
|  |  | DAAM2 |
|  |  | FYCO1 |
|  |  | GOLGB1 |
|  |  | LRRC7 |
|  |  | ATP8A1 |
|  |  | ASPM |
|  |  | DMXL2 |
|  |  | LAMA3 |
|  |  | CCDC88A |
|  |  | TPM2 |
|  |  | KIF15 |
|  |  | SYNE1 |
|  |  | ANK3 |
|  |  | DNAJC13 |
|  |  | DNAH11 |
|  |  | GOLGA5 |
|  |  | FAT3 |

| **I B. Comparison between sepsis survivors and non-survivors at day 7 in HAP (D7 S x D7 NS)** | | |
| --- | --- | --- |
| 20 proteins included exclusively in "D7 S": | 23 proteins included exclusively in "D7 NS": | 40 common proteins in "D7 S" and "D7 NS": |
| C3 | APOA1 | FGA |
| APOB | SAA1 | SERPINA3 |
| HBB | SAA2 | LRG1 |
| APOE | FGG | F2 |
| A1BG | CRP | PON1 |
| HBD | POTEF | VTN |
| ITIH1 | POTEE | HP |
| CFB | APOC1 | GSN |
| APOL1 | PLCH1 | APOA4 |
| DST | SAA4 | HPR |
| DNAH8 | SERPING1 | CPN1 |
| SERPINA6 | GPX3 | KRT76 |
| BDP1 | AZGP1 | HBA1 |
| FRAS1 | FN1 | ITIH2 |
| GOLGB1 | PROS1 | SERPIND1 |
| SYNE1 | CROCC | C8A |
| DNAJC13 | KIF7 | PIK3C2G |
| DNAH11 | KIF20B | HLTF |
| GOLGA5 | PCNT | C8B |
| MUC16 | MKI67 | KMT2C |
|  | OBSCN | DNAH5 |
|  | SPTBN5 | JAK1 |
|  | MCF2L2 | DAAM2 |
|  |  | FYCO1 |
|  |  | FLNB |
|  |  | PRPF8 |
|  |  | LRRC7 |
|  |  | ATP8A1 |
|  |  | HGFAC |
|  |  | CTTNBP2 |
|  |  | ASPM |
|  |  | DMXL2 |
|  |  | LAMA3 |
|  |  | TPM2 |
|  |  | KIF15 |
|  |  | ANK3 |
|  |  | SPTBN1 |
|  |  | EVPL |
|  |  | AHNAK2 |
|  |  | FAT3 |

| **I C. Comparison between HAP survivors and non-survivors at day 0 and day 7** | | | | |
| --- | --- | --- | --- | --- |
| 27 common proteins in "D0 S", "D7 S", "D0 NS" and "D7 NS": | 9 proteins included exclusively in  "D0 S": | 3 proteins included exclusively in "D7 S": | 13 proteins included exclusively in "D0 NS": | 10 proteins included exclusively in "D7 NS": |
| FGA | APOC2 | ITIH1 | CP | APOA1 |
| SERPINA3 | APOC3 | CFB | TF | POTEF |
| LRG1 | FBLN1 | BDP1 | APOA2 | POTEE |
| F2 | PZP |  | DNAH17 | SERPING1 |
| PON1 | C6 |  | CEP350 | GPX3 |
| HP | KNG1 |  | ELMO1 | AZGP1 |
| APOA4 | PNPLA8 |  | LBP | FN1 |
| HPR | TTN |  | ORM2 | PROS1 |
| KRT76 | TPR |  | LYZ | CROCC |
| ITIH2 |  |  | LTF | KIF20B |
| SERPIND1 |  |  | NIPBL |  |
| PIK3C2G |  |  | SPAG17 |  |
| HLTF |  |  | WDR87 |  |
| KMT2C |  |  |  |  |
| DNAH5 |  |  |  |  |
| JAK1 |  |  |  |  |
| DAAM2 |  |  |  |  |
| FYCO1 |  |  |  |  |
| LRRC7 |  |  |  |  |
| ATP8A1 |  |  |  |  |
| ASPM |  |  |  |  |
| DMXL2 |  |  |  |  |
| LAMA3 |  |  |  |  |
| TPM2 |  |  |  |  |
| KIF15 |  |  |  |  |
| ANK3 |  |  |  |  |
| FAT3 |  |  |  |  |

| **II A. Comparison between CAP and HAP survivor septic patients at day 0 (CAP D0S x HAP D0S)** | | |
| --- | --- | --- |
| 38 proteins included exclusively in "CAP D0S": | 35 proteins included exclusively in "HAP D0S": | 26 common proteins in "CAP D0S" and "HAP D0S": |
| SERPINA1 | C3 | FGA |
| FGB | APOB | SAA1 |
| CPN1 | APOE | SAA2 |
| APOA2 | FBLN1 | SERPINA3 |
| GSN | PZP | APOA4 |
| APOD | KRT76 | HP |
| LBP | ITIH2 | LRG1 |
| OBSCN | APOL1 | FGG |
| ORM1 | C6 | HPR |
| MYO5B | KNG1 | F2 |
| SMC3 | DST | APOC3 |
| RNF213 | PIK3C2G | CRP |
| KIAA1109 | HLTF | PON1 |
| NF1 | PLCH1 | APOC2 |
| ORM2 | SAA4 | APOC1 |
| KLKB1 | C8B | DNAH8 |
| DNAH12 | LRRK2 | LAMA3 |
| FSIP2 | SERPINA6 | DNAH5 |
| SVEP1 | JAK1 | SERPIND1 |
| COL6A3 | FRAS1 | SYNE1 |
| NUMA1 | DAAM2 | TTN |
| TRANK1 | FYCO1 | GOLGB1 |
| MYO5A | LRRC7 | DMXL2 |
| PLEC | ATP8A1 | DNAH11 |
| GCC2 | CCDC88A | KMT2C |
| MYO7A | TPM2 | ASPM |
| SPTAN1 | KIF15 |  |
| DNAH10 | ANK3 |  |
| HEXIM1 | PNPLA8 |  |
| MYH9 | DNAJC13 |  |
| ALMS1 | SPTBN1 |  |
| KIF27 | GOLGA5 |  |
| SPTBN4 | TPR |  |
| SRCAP | MUC16 |  |
| ATM | FAT3 |  |
| CENPE |  |  |
| EVPL |  |  |
| MYH13 |  |  |

| **II B. Comparison between CAP and HAP non-survivor patients at day 0 (CAP D0NS x HAP D0NS)** | | |
| --- | --- | --- |
| 35 proteins included exclusively in "CAP D0NS": | 42 proteins included exclusively in "HAP D0NS": | 33 common proteinss in "CAP D0NS" and "HAP D0NS": |
| SERPINA1 | C3 | FGA |
| FGB | CP | SAA1 |
| FGG | TF | SAA2 |
| TTR | APOB | SERPINA3 |
| APOC3 | APOE | APOA4 |
| APOC2 | A1BG | HP |
| QSOX1 | CPN1 | LRG1 |
| ORM1 | KRT76 | HPR |
| ACTB | ITIH2 | F2 |
| MYO5B | APOL1 | HBB |
| C1R | PIK3C2G | CRP |
| SERPING1 | HLTF | PON1 |
| PROS1 | PLCH1 | APOA2 |
| DYSF | DNAH17 | GSN |
| NF1 | CEP350 | HBD |
| PIBF1 | ELMO1 | LBP |
| AFM | SAA4 | APOC1 |
| DNAH12 | LRRK2 | DNAH8 |
| FSIP2 | JAK1 | LAMA3 |
| SVEP1 | FRAS1 | DNAH5 |
| SHROOM3 | DAAM2 | DST |
| COL6A3 | FYCO1 | SERPIND1 |
| NUMA1 | FLNB | HBA1 |
| TRANK1 | PRPF8 | GOLGB1 |
| SYNE2 | LYZ | DMXL2 |
| GCC2 | LRRC7 | DNAH11 |
| SPTAN1 | ATP8A1 | ORM2 |
| DNAH10 | KIF7 | ANK3 |
| HEXIM1 | CCDC88A | KMT2C |
| MYH9 | TPM2 | PCNT |
| ALMS1 | KIF15 | NIPBL |
| KIF27 | SYNE1 | ASPM |
| SBF1 | MKI67 | EVPL |
| SRCAP | OBSCN |  |
| MDN1 | LTF |  |
|  | SPAG17 |  |
|  | SPTBN5 |  |
|  | WDR87 |  |
|  | DNAJC13 |  |
|  | GOLGA5 |  |
|  | MCF2L2 |  |
|  | FAT3 |  |

| **II C. Comparison between CAP and HAP survivor septic patients at day 7 (CAP D7S x HAP D7S)** | | |
| --- | --- | --- |
| 57 proteins included exclusively in "CAP D7S": | 38 proteins included exclusively in "HAP D7S": | 22 common proteins in "CAP D7S" and "HAP D7S": |
| SERPINA1 | C3 | FGA |
| SAA1 | APOB | SERPINA3 |
| SAA2 | APOE | APOA4 |
| FGB | A1BG | HP |
| CP | VTN | LRG1 |
| FGG | ITIH1 | HPR |
| CLU | CFB | F2 |
| HPX | CPN1 | HBB |
| CRP | KRT76 | PON1 |
| APOA2 | ITIH2 | GSN |
| LBP | APOL1 | HBD |
| JCHAIN | C8A | LAMA3 |
| QSOX1 | DST | DNAH5 |
| APOC1 | PIK3C2G | SERPIND1 |
| C6 | DNAH8 | HLTF |
| OBSCN | C8B | HBA1 |
| ORM1 | SERPINA6 | GOLGB1 |
| ACTB | BDP1 | DMXL2 |
| MYO5B | JAK1 | DNAH11 |
| CFHR2 | FRAS1 | KMT2C |
| TTN | DAAM2 | ASPM |
| GPX3 | FYCO1 | EVPL |
| CEP290 | FLNB |  |
| CNTRL | PRPF8 |  |
| SACS | LRRC7 |  |
| RNF213 | ATP8A1 |  |
| NF1 | HGFAC |  |
| ACTA1 | CTTNBP2 |  |
| SVEP1 | TPM2 |  |
| POTEI | KIF15 |  |
| POTEE | SYNE1 |  |
| POTEF | ANK3 |  |
| SYCP2 | DNAJC13 |  |
| COL6A3 | SPTBN1 |  |
| CCDC88C | GOLGA5 |  |
| SYNE2 | AHNAK2 |  |
| PCNT | MUC16 |  |
| PCM1 | FAT3 |  |
| GCC2 |  |  |
| BPTF |  |  |
| MYO7A |  |  |
| DNAH10 |  |  |
| HEXIM1 |  |  |
| NIPBL |  |  |
| MYH9 |  |  |
| KIF27 |  |  |
| SPTBN4 |  |  |
| SBF1 |  |  |
| SRCAP |  |  |
| MYO9A |  |  |
| ATM |  |  |
| TRPM2 |  |  |
| CENPE |  |  |
| BOD1L1 |  |  |
| MDN1 |  |  |
| MYH13 |  |  |
| LAMA4 |  |  |

| **II D. Comparison between CAP and HAP non-survivor patients at day 7 (CAP D7NS x HAP D7NS)** | | |
| --- | --- | --- |
| 48 proteins included exclusively in "CAP D7NS": | 36 proteins included exclusively in "HAP D7NS": | 27 common proteins in "CAP D7NS" and "HAP D7NS": |
| FGB | APOA1 | FGA |
| CP | CPN1 | SAA1 |
| HBB | KRT76 | SAA2 |
| APOA2 | ITIH2 | SERPINA3 |
| A1BG | POTEF | APOA4 |
| HBD | POTEE | HP |
| CFHR1 | PIK3C2G | VTN |
| LBP | PLCH1 | LRG1 |
| JCHAIN | SAA4 | FGG |
| C9 | C8B | HPR |
| MYO5B | GPX3 | F2 |
| APOL1 | AZGP1 | CRP |
| CFHR2 | FN1 | PON1 |
| TTN | PROS1 | GSN |
| CFI | JAK1 | APOC1 |
| GOLGB1 | DAAM2 | C8A |
| CNTRL | FYCO1 | OBSCN |
| SERPINA4 | FLNB | LAMA3 |
| NF1 | PRPF8 | DNAH5 |
| LAMC2 | CROCC | SERPIND1 |
| DNAH11 | LRRC7 | SERPING1 |
| PIBF1 | ATP8A1 | HLTF |
| KLKB1 | KIF7 | HBA1 |
| ACTA1 | HGFAC | DMXL2 |
| SVEP1 | CTTNBP2 | KMT2C |
| GOLGA4 | KIF20B | ASPM |
| COL6A3 | TPM2 | EVPL |
| NUMA1 | KIF15 |  |
| SYNE2 | ANK3 |  |
| F13A1 | PCNT |  |
| PCM1 | MKI67 |  |
| CCDC88A | SPTBN5 |  |
| GCC2 | SPTBN1 |  |
| BPTF | MCF2L2 |  |
| ZFHX4 | AHNAK2 |  |
| SPTAN1 | FAT3 |  |
| HEXIM1 |  |  |
| NIPBL |  |  |
| MYH9 |  |  |
| KIF27 |  |  |
| SPTBN4 |  |  |
| SBF1 |  |  |
| SRCAP |  |  |
| ATM |  |  |
| TRPM2 |  |  |
| BOD1L1 |  |  |
| LYST |  |  |
| MDN1 |  |  |
