# Supplementary material for: Lipid metabolism impairment in patients with sepsis secondary to hospital acquired pneumonia, a proteomic analysis
Source: Clin Proteomics. 2019 Jul 16;16:29. doi: 10.1186/s12014-019-9252-2 (PMC6631513; doi:10.1186/s12014-019-9252-2)
Supplement: Supplementary file 3 — Additional file 3. IPA canonical pathway analysis in septic patients. A refers to altered canonical pathway in D0 survivors; B refers to D0 non-survivors; C refers to D7 survivors; and D refers to D7 non-survivors. Enriched canonical pathways were identified from the IPA library using Fisher’s exact test adjusted for multiple hypothesis testing with the Benjamini- Hochberg correction. [file 12014_2019_9252_MOESM3_ESM.docx]

**
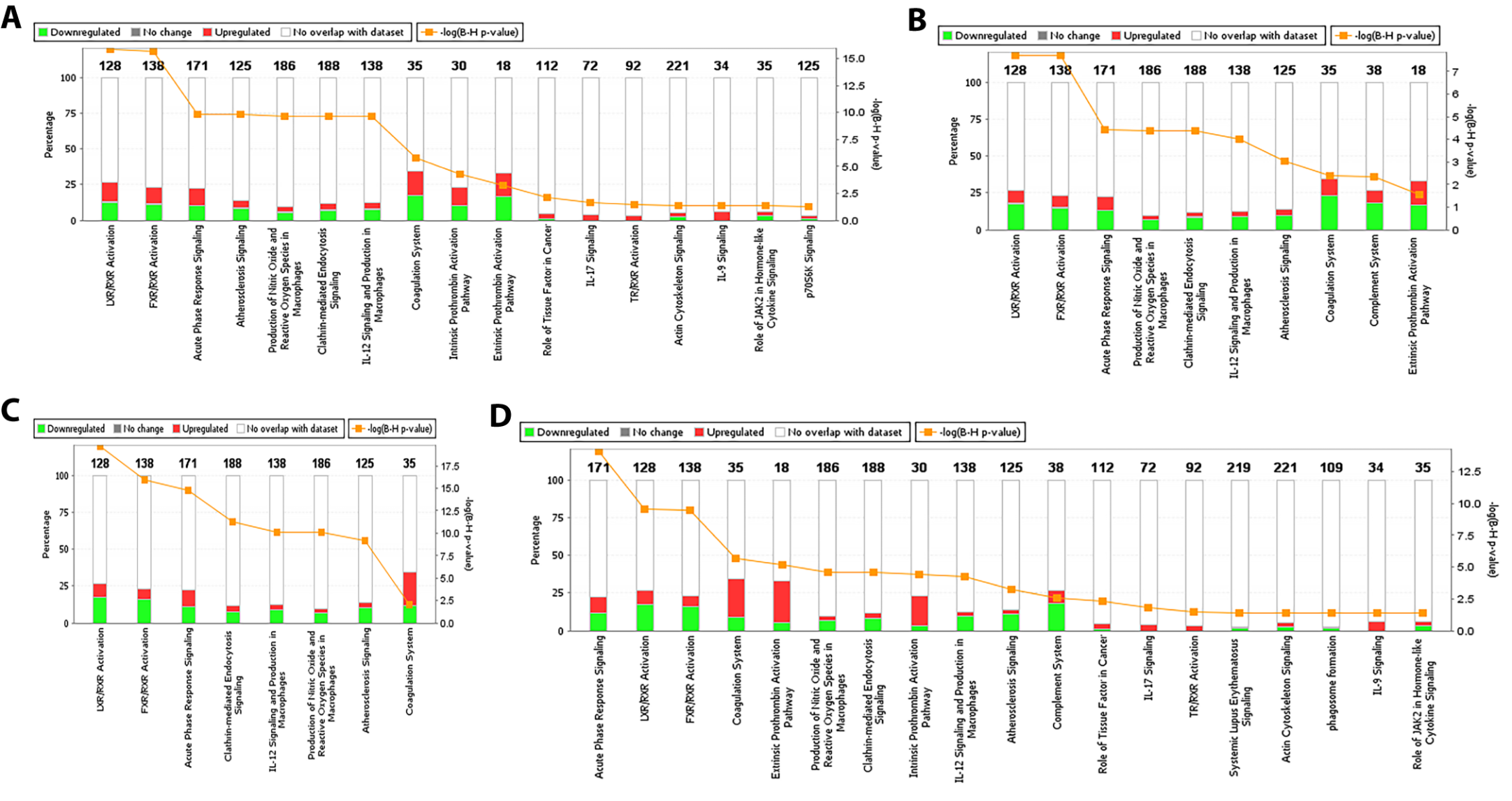
**

**Additional File 3. IPA canonical pathway analysis in septic patients.** A refers to altered canonical pathway in D0 survivors; B refers to D0 non-survivors; C refers to D7 survivors; and D refers to D7 non-survivors. Enriched canonical pathways were identified from the IPA library using Fisher’s exact test adjusted for multiple hypothesis testing with the Benjamini- Hochberg correction.
