# Supplementary material for: Lipid metabolism impairment in patients with sepsis secondary to hospital acquired pneumonia, a proteomic analysis
Source: Clin Proteomics. 2019 Jul 16;16:29. doi: 10.1186/s12014-019-9252-2 (PMC6631513; doi:10.1186/s12014-019-9252-2)
Supplement: Supplementary file 4 — Additional file 4. Functional protein interaction network for septic patients. The bold symbols represent the proteins identified in our study with their functions. The green arrow shows decreased expression and red arrow show increased expression compared with healthy volunteers. [file 12014_2019_9252_MOESM4_ESM.docx]

**Additional File 4. Functional protein interaction network for septic patients.** The bold symbols represent the proteins identified in our study with their functions. The green arrow shows decreased expression and red arrow show increased expression compared with healthy volunteers.

D0 - survivor

| **ID** | **Molecules in Network** | **Score** | **Focus Molecules** | **Top Diseases and Functions** |
| --- | --- | --- | --- | --- |
| 1 | **↓APOA4, ↓APOB, ↓APOC1, ↓APOC2 ↓APOC3, ↓APOAL1,** **↑CCDC88A,** **↑CRP,** ELASTASE, ERK1/2, **↓FBLN1,** **↑FGA, ↑FGG,** Fibrin, FXR ligand-FXR-Retinoic acid-RXR**α**, GPIIB-IIIA, HDL, HDL-cholesterol, hemoglobin, **↑HP, ↑HPR, ↑LAMA3,** LDL-cholesterol, NCOR-LXR-Oxysterol-RXR-9 cis RA, Nos, Nrh1, **↓PON1,** SAA, **↑SAA1, ↑SAA2, ↓SAA4, ↓SERPINA6,** Stat3-Stat3, VLDL-Cholesterol | 42 | 19 | Lipid Metabolism, Molecular Transport, Small Molecule Biochemistry |
| 2 | ADH4, ADH1B, **↑ASPM, ↓C8A, ↓C8B, ↓C8G,** CBR3, **↑CCDC88A,** CD2AP, **↑DMXL2,** EOMES, **↓FAT3, ↑FGA, ↑FYCO1, ↑GOLGB1,** HNF1A, Ifnz (includes others), IL11RA, KDM1A, **↑KRT76,** MON1B, **↑MUC16,** PTGER2, **↓PZP,** RASSF8, RRAD, SLC12A7, SOD2, **↑SPTBN1,** STAT3, TGFB1, TLR7, TRPM2, YWHAE, ZNF217 | 24 | 12 | Cancer, Organismal Injury and Abnormalities, Reproductive System Diseases |
| 3 | Akt, Alpha Actinin, **↑ANK3,** AP1, **↓APOE,** chymotrypsin, Collages (s), **↑DST, ↓F2,** F actin, Fibrinogen, IgG, IL1, IL12 (complex), **↑JAK1,** Kallikrein, **↑KNG1,** Laminin, LDL, **↑LRG1, ↑LRRC7,** Mapk, Mek, Pdgf (complex), P13K (complex), **↑PIK3C2G,** Pro-inflammatory Cytokine, Serine Protease, **↑SERPINA3, ↓SERPIND1,** Shc, Sos, Tgf beta, trypsin, **↓TTN** | 22 | 12 | Lipid Metabolism, Small Molecule Biochemistry, Cardiovascular System Development and Function |

D0- non-survivor

| **ID** | **Molecules in Network** | **Score** | **Focus Molecules** | **Top Diseases and Functions** |
| --- | --- | --- | --- | --- |
| 1 | **↑A1BG, ↑APOA2, ↓APOA4, ↓APOB, ↓APOC1, ↓APOE ↓APOAL1,** **↑CCDC88A,** **↑CP,** **↑CRP,** ERK1/2, Ferritin, **↑FGA, ↑HBA1/HBA2, ↑HBB, ↑HBD,** HDL, HDL-cholesterol, hemoglobin, **↑HP, ↑HPR, ↑LAMA3, ↑LBP,** Nos, **↓ORM2,** **↓PON1,** Proinflammatory cytokine, SAA, **↑SAA1, ↑SAA2, ↓SAA4,** Stat3-Stat3, **↓TF,** VLDL, VLDL-Cholesterol | 56 | 24 | Lipid Metabolism, Molecular Transport, Small Molecule Biochemistry |
| 2 | AAR2, ACBD3, CCDC8, **↑CEP350,** CUL3, DLG4, **↑DNAH1,** DNAH2, DNAH3, **↑DNAH5,** DNAH6, **↑DNAH8,** DNAH9, DNAH10, **↑DNAH11,** DNAH12, DNAH14, **↑DNAH17,** DNAI1, DNAI2, DNAI4, DNAIL1, **↑DST,** DYNLT1, ERG, **↑FRAS1, ↑GOLGB1, ↓KIF7, ↑KRT76, ↑LRRC7,** NRCAM, **↓PRPF8, ↑SPTBN5,** TCTE3 | 26 | 13 | Development Disorder, Hereditary Disorder, Respiratory Disease |
| 3 | 15-hydroxyeicosatetraenoic acid, ADGRB1, **↑ASPM, ↑ATP8A1,** c-Src, Ca2+, CDC42EP3, CKMT1A/CKMT1B, **↓DAAM2, ↑DMXL2,** EGFR, EOMES, **↑EVPL, ↓FAT3, ↑FYCO1, ↓GOLGA5, ↓HLTF,** HNF4A, IL13, IL11RA, **↓ITIH2, ↓ITIH3, ↓MCF2L2,** MDM2, MON1B, MRT04, NDUFA1, **↑PLCH1,** S100A12, SPINT2, SULT1C2, TAOK3, TGFB1, TMEM30A, TPM4 | 22 | 12 | Skeletal and Muscular System Development and Function, Cellular Development, Cell Morphology |

D7- survivor

| **ID** | **Molecules in Network** | **Score** | **Focus Molecules** | **Top Diseases and Functions** |
| --- | --- | --- | --- | --- |
| 1 | **↑A1BG, ↓APOA4, ↓APOB, ↓APOE ↓APOAL1,**  chymotrypsin, Collagen(s), **↓CPN1,** ERK1/2, **↑FGA,**  Fibrin, Fibrinogen, GPIIB-IIIA, **↑HBA1/HBA2, ↑HBB, ↑HBD,** HDL, HDL-cholesterol, hemoglobin, **↑HP, ↑HPR,** Iti, **↓ITIH1, ↓ITIH2,** **↑LAMA3,** LDL, LDL-Cholesterol, NADPH oxidase, **↑PIK3C2G,** **↓PON1,** Proinflammatory cytokine, Serine Protease, **↑SERPINA3, ↓SERPIND1, ↓VTN** | 43 | 20 | Cardiovascular Disease, Connective Tissue Disorders, Hematological Disease |
| 2 | 26s Proteasome, Akt, **↑ANK3, ↓C8B,** caspase, **↓CFB, ↓DNAJC13, ↑DST, ↓F2,** F actin, **↓FLNB, ↓GSN, ↓HGFAC,** Hsp70, Hsp90, IL1, Immunoglobulin, Insulin, Interferon alpha, **↑JAK1, Jnk, ↑KIF15, ↑LRG1,** Mac, p85(pik3r), Pdgf(complex), P13K(complex), Pka, Sos, **↑SPTBN1,** STAT, Tgf beta, **↑TPM2,** Tropomyosin, Vegf | 27 | 14 | Cellular Assembly and Organization, Developmental Disorder, Hereditary Disorder |
| 3 | **↑ASPM,** beta-estradiol, **↑CTTNBP2,** CUL3, DLG4, **↑DMXL2,** **↑DNAH1,** DNAH2, DNAH3, **↑DNAH5,** DNAH6, **↑DNAH8,** DNAH9, DNAH10, **↑DNAH11,** DNAH12, DNAH14, **↑DNAH17,** DNAI1, DNAI2, DNAL1, DNAIL4, DNALI1, DYNLT1, **↑FRAS1,** GRIP2, **↑KRT76, ↑LRRC7,** PPP2RIA, **↓PRPF8, ↓SERPINA6, ↓SYNE1,** TCP1, TCTE3, YWHAZ | 24 | 12 | Developmental Disorder, Hereditary Disorder, Respiratory Disease |

D7 – non-survivor

| **ID** | **Molecules in Network** | **Score** | **Focus Molecules** | **Top Diseases and Functions** |
| --- | --- | --- | --- | --- |
| 1 | **↓APOA1, ↓APOA4, ↓APOC1,** chymotrypsin, complement component 1, **↓CPN1,** creatine kinase, **↑CRP,** elastase, ERK1/2, **↑FGA, ↑FGG,** Fibrin, Fibrinogen, GPIIB-IIIA, Growth hormone, HDL, HDL- cholestrole, hemoglobin, **↑HP, ↑HPR,** **↓LAMA3,** LDL-cholesterole, **↓PON1,** PRKAA, Pro-inflammatory Cytokine, **↑PROS1,** SAA, **↑SAA1, ↑SAA2, ↓SAA4, ↑SERPING1,** Stat3-Stat3, VLDL-cholesterole, **↓VTN** | 35 | 17 | Protein Synthesis, Metabolic Disease, Cell –To- Cell Signaling And Interaction |
| 2 | Akt, **↑ANK3,** **↓AZGP1,** Collagen type IV, Collagen(s), ERK, **↓F2,** F Actin, **↓FLNB, ↓FN1,** Focal adhesion kinase, **↓GSN, ↑HBA1/HBA2, ↓HGFAC,** IgG Immunoglobulin, Integrin, **↓ITIH2,** Laminin, LDL, **↑LRG1,** NAPDH oxidase, **↑OBSCN,** Pak, Pdgf(complex), PId, Rac, Rock, **↑SERPINA3, ↓SERPIND1,** Sos, **↑SPTBN1,** Tqf beta, **↑TPM2,** Tropomyosin | 30 | 15 | Cellular Assembly and Organization, Hematological Disease, Hereditaory Disease |
| 3 | **↓AHNAK2,** Alpha actin, **↑ASPN, ↑ATP8A1,** BDKRB1, CA2+, CASP3, CD2AP, CNN3, **↑CROCC,** CTTN, **↓CTTNBP2,** delta-aminolevulinic acid, DOK5, EGFR, EOMES, ESM1, **↑EVPL, ↓FAT3,** IL20RA, K Channel, MAPK1, **↓MCF2L2,** MGEA5, MTSS1, **↑PLCH1,** RNF152, S100A12, SLC12A7, SPINT2, ST14, TGFB1, TNS4, TTC27, UCK2 | 17 | 9 | Cellular Movement, Cellular Development, Embryonic Development |
