# Supplementary material for: Lipid metabolism impairment in patients with sepsis secondary to hospital acquired pneumonia, a proteomic analysis
Source: Clin Proteomics. 2019 Jul 16;16:29. doi: 10.1186/s12014-019-9252-2 (PMC6631513; doi:10.1186/s12014-019-9252-2)
Supplement: Supplementary file 5 — Additional file 5. Functional analysis curated by Ingenuity Pathway Analyses. Prediction of altered functions based on activation Z-score. A score lower than -2 or higher than 2 predicts decreased or increased activation for each function. S: survivor. NS: non-survivor. [file 12014_2019_9252_MOESM5_ESM.docx]

|  | **Categories** | **Diseases or functions annotations** | **p value** | **Activation Z-score** | **molecules** | **Predicted activation** |
| --- | --- | --- | --- | --- | --- | --- |
| **D0**  **S** | Lipid metabolism, molecular transport, small molecule biochemistry | Transport of phospholipid | 3.01E-9 | **-2.41** | 7 | Decreased |
|  |  | Efflux of phospholipid | 4.29E-8 | **-2.20** | 5 | Decreased |
|  |  | Transport of lipid | 2.32E-9 | **-2.03** | 11 | Decreased |
| **D0 NS** | Lipid metabolism, small molecule biochemistry | Fatty acid metabolism | 1.21E-5 | -1.92 | 13 |  |
|  | Lipid metabolism, molecular transport, small molecule biochemistry | Transport of phospholipid | 3.28E-7 | -1.96 | 6 |  |
| **D7**  **S** | Lipid metabolism, molecular transport, small molecule biochemistry | Transport of lipid | 4.03E-6 | **-2.36** | 8 | Decreased |
|  |  | Transport of steroid | 2.03E-5 | **-2.16** | 6 | Decreased |
| **D7 NS** | Carbohydrate metabolism | Binding of carbohydrate | 1.74E-5 | **-2.21** | 5 | Decreased |
|  | Lipid metabolism, molecular transport, small molecule biochemistry | Transport of phospholipid | 3.61E-6 | -1.92 | 5 |  |

**Additional File 5. Functional analysis curated by Ingenuity Pathway Analyses**. Prediction of altered functions based on activation Z-score. A score lower than -2 or higher than 2 predicts decreased or increased activation for each function. S: survivor. NS: non-survivor.
